# Selections and Abstracts

**Published:** 1897-03

**Authors:** 


### SELECTIONS AND ABSTRACTS.

---

### THE PASSING OF THE REFLEX.*

I do not believe that medical men generally realize how entirely our pathology is bound up and interwoven with conceptions of the reflexes. Three thousand years ago Hippocrates suggested that hysteria was a disease that was dependent upon disorder of the womb, and at the present day the physician cannot rid himself of the idea that the hysterical woman must necessarily have some disorder of that organ; yet facts show that true hysteria occurs in men and in children of both sexes, and there is plenty of evidence to show that women with their pelvic cavities nearly emptied can still have a typical hysterical attack. Marshall Hall, who was the great high priest of the reflexes in the early part of this century,

*Abstract of an address upon the "Reflex Origin of Nervous Diseases," by Dr. Chas. L. Dana, New York. From the *Postgraduate.*

stated that the principal causes of eccentric epilepsy were only three: "The presence of indigestible food in the stomach, the presence of morbid matters in the intestine, and uterine irritation." The profession is still profoundly under the influence of such statements.

The theory of reflex action was first distinctly recognized by Des Cartes, but it was first brought into medicine by Dr. Robert Whytt over one hundred years ago. The physiology of reflex action, and the importance of it in the phenomena of the body, were most diligently worked out in the years succeeding Whytt, by Prochaska, in the last century, and by Mayo, Marshall Hall, Muller, and Ludwig in this century. It is now almost half a century since the facts regarding the importance of the reflex loop were given to science. Since then the application of these has been given to the practical art of medicine, with the ever-widening circles of the alluring but delusive therapeutics. When the history of medicine of this century comes to be written, the reflex loop will be found to be filled with the dangling remains of unsuccessful therapeutical and surgical endeavors.

There was once a distinguished surgeon, Dr. Robert Battey, who conceived the theory that the normal ovaries were the starting point of a large array of chronic ailments, and he reported some fourteen cases of epilepsy cured by removing the normal organ. Not more than two decades ago it was believed that paraplegia could be caused by reflex irritations from the intestines, the bladder, and other organs. Literature of the last thirty years has been filled with the histories of reflex neuroses from the ear, from the eye, from the teeth, the nose, the stomach, and every organ which could be reached by the instrument of the surgeon or the manipulation of the physician. We read of reflex amblyopia, reflex deafness, or reflex epilepsy, paraplegia, chorea, tetanus, aphasia, neuralgia, and headache.

It is my desire to-night to present to you what I conceive to be the true position the physician should take in regard to the importance of these reflex impulses, and you will see that, while I do not ignore their value, I want especially to impress upon you the importance of attacking in your therapeutics the centers of the disorder, rather than nibbling away at its outer edge.

My first and perhaps my chief general proposition is this: that there are no reflex constititutional neuroses. By this I mean that those neuroses which are general in character, and which are classed by systematic writers as *constitutional*, are not dependent upon reflex irritants, and cannot be cured by attacking these. Such neuroses are, in particular chorea, the functional spasms, such as convulsive tic, epilepsy, paralysis agitans; even hysteria of the true, the major type, as I have described it in my lectures here and in my text-book, is never dependent upon a remote local irritation. Spinal irritation, in its typical form, and the neuralgias of a constitutional or degenerative type, such, in particular, as migraine in early life, and the tic douloreux of middle life, may be added to the list.

*Chorea.*—There are very few physicians who still maintain that chorea is a reflex neurosis. A certain limited number, however, do assert that by proper treatment of the eye muscles the chorea can be cured, and particularly those forms of chorea which are more properly classed with the convulsive tics, or which are often known as "habit" and "chronic" choreas. So far as these spasmodic movements are concerned, all that eye and nasal irritation can cause is a certain degree of twitching, that is to say, those muscles which are intimately associated with the movements of the eye may be reflexly kept irritated and in a state of mobile spasm by eye disease.

*Epilepsy.*—As for epilepsy, it will, I think, be a great relief to some, and a great shock to the traditions of others, to be assured that true epilepsy is always a central disease. It is not caused essentially by reflex irritation, and it is not cured by treatment of the eyes, or nose, or teeth, or stomach, or bowels. It is not cured by taking out the ovaries, or by removal of the uterus, or readjustment of the kidneys, nor even by phimosis, or by removal of the appendix. You will find in literature a good many cases in which epilepsy is alleged to have been caused by some scar or bite, by the presence of worms in the intestine, or by a blow upon some part of the body, and there are cases in which cures have been reported from cutting off the scar, removing the worms or the offending body or organ. It is very interesting and instructive, however, to note that these cases increase in number the further we go back in the literature of medicine, and that with each succeeding year their number in current literature diminishes. For my own part, in a list of epileptic cases which numbers over three hundred, I have never come across any such instance.

It will, I think, be found that nearly all cases of remarkable cures from removing some local trouble are either temporary, or that the central trouble is not true epilepsy, or has not fully established itself as such.

As to the reaction of epilepsy and eye-strain, it may be known to you that several years ago a committee of the New York Neurological Society examined into this subject, their investigations extending over two years. They reported that they could find no evidence that treatment of muscular or refractive defects of the eyes caused either epilepsy or chorea.

Noyes, who views this subject with a liberal mind, says in his text-book, that "in a few cases eye-strain may have been an exciting cause, but there has been behind it a deeper lesion of the nervous system."

*Neurasthenia.*—There have been periods in the history of this disorder when all neurasthenia was thought to be due either to eye-strain or uric acid; there was a time also when hypochondriasis was thought to be a matter of constipation and the liver. It is not worth while taking up your time now in refuting these views. For, while it is acknowledged that they may all be factors in breeding nervous symptoms, there is something behind them that is more important, and that is a naturally defective or an exhausted nerve-center. I have seen neurasthenics vastly improved for months by tenotomy of the eye-muscles, and by washing out the stomach, and careful attention to diet; but when the condition is anywise a fixed one, these measures alone are ineffective.

A great deal of help can be given sometimes in neurotic and neuralgic women by scraping the uterus, sewing up tears and replacing the dislocated organ. But often it is the rest in bed and relief from care, as much as the operation, which helps when help is really given. As for the results of more serious surgery, Playfair cites a number of cases in point, and asserts that the removal of the appendages "is not a legitimate procedure in cases of purely functional neuroses."

*Spinal Irritation.*—Women with pain in the back are often relieved by treatment of the pelvic organs. There is a disease, however, known as spinal irritation, in which the whole back is painful and sensitive; the patients are nervous, neuralgic, have headaches, are easily exhausted, and show signs of hysteria and neurasthenia. Here the trouble is constitutional in part and local in part, but it is not a uterine, or ovarian, or gastric, or renal reflex. These patients are helped by sthenic measures, gymnastics, braces and counter-irritation. They need not have their kidneys pinned up or their appendix resected, the clitoris freed from adhesions, or the uterus and its ovarian allies removed.

I admire immensely the surgical skill and daring of the present day. Its effect, on the whole, has been enormously beneficial. But I would remove from its aggressive enthusiasm the reflex arc. This arc is a physiological and anatomical fact of much intrinsic beauty and vital importance; but we have made of it a rainbow of seductive and unwarrantable promise, with an occasional pot of gold at one end. The new woman is especially imperiled. She used to have to watch the hymen; now she must look out for the appendix, the adnexæ and the mobility of her kidney with as much care as if they were her cardinal virtues.

I have, perhaps, spoken too lightly of the importance of peripheral irritation, of a loaded stomach, an astigmatic eye, a displaced uterus. Pray do not misunderstand me; I wish only to make you feel that the man is greater than his parts; that to study his general condition is better than to squint into any particular viscus. I have seen migraine relieved by glasses, vertigo cured by removing wax from the ear, intense spinal pains relieved by replacing the uterus. I have seen wretched neurasthenic women made over by gynecological operations, and I render in full my sense of the obligation of medicine to its special workers. But I repeat again that fixed diseases are never cured by treatment of reflex irritation. There are reflex pains, reflex spasms, reflex mental disturbances there are reflex symptoms, many and important, but there are no reflex diseases in any true and serious sense of the word. If I have succeeded in impressing this upon you, I believe you will be better physicians and more learned and clear-sighted men.

---

### THE CONFESSIONS OF A COCAINIST.

The *Journal of Inebriety* for January reproduces part of an article in the Australasian *Medical Gazette* by Dr. Springthorpe, which relates the history and sensations of a cocaine habitué. The patient was a medical man of "great mental endowments and uncommon training." He first began taking cocaine in one-grain doses, while serving (1885) in the German army, and he noticed that he could complete his long marches and arrive at quarters fresh, and neither tired, nor hungry, nor thirsty. While practicing medicine four years later he was summoned to a confinement case "when stiff and unable to move with lumbago." "Remembering well the effect of the cocaine I took a syringe (one-half centigram) combined with morphine, and two minutes afterwards one centigram. Five minutes later I was ready to start two miles in a snow storm. That night in November, 1889, settled my future." He took cocaine and morphine from that time forward, and soon was able to take as much as eighty or a hundred grains of the former daily. He says: "The first feeling a cocainist has is an indescribable excitement to do something great; to leave a mark. This disappears as rapidly as it came and every part of the body seems to cry out for a new syringe. Then his hearing becomes enormously increased, and soon every sound begins to be a remark about himself. He begins to carry on a solitary life, his only companion being his beloved syringe. . . . After a relatively short time begins the 'hunting for the cocaine bug.' One imagines that worms and similar things are moving in his skin. If you touch them they run away, only to reappear. These worms are projected only on the cocainist's own person or clothing, not on those of other people, and not on clothes brought clean from the laundry. . . . Other dreadful hallucinations I had in thousands, all of a persecuting character, and frightening the life out of me so long as the effects of the drug lasted. . . .

About that time I bought three large dogs, thinking they would protect me; but one night I found out that they were all talking about me, so I stood up and shot one of them with a revolver. I think this was the most dreadful night of my life—I standing on the table, with an Indian dagger and a syringe on the ground; one dog going to die, and two other dogs roaring and groaning aloud, reproachfully looking at me. I stood the night on the table, till the arrival of my wardsman, who hardly risked to enter the room."

Not frightened enough by these and similar experiences, he went on taking more and more, and in a year's time he was "placed under restraint," and his habit had cost him about $8,000. His experiences had embraced the whole gamut of wretchedness and shame, and included both hospital and jail.

He summarizes the physiological effects of the drug as follows:

"The cocainist early loses all appetite for solid food, but likes sweets, jellies, and cakes. Diarrhea is soon produced, and immediate evacuation often follows big injections. Upon the muscular system the drug, as is generally recognized, acts as a most powerful stimulant for either single or continued effort. . . . It increases also the number of the respiratory and of the cardiac contractions (with vascular dilatation), as well as the quantity of urine (with large or repeated small doses, incontinence follows), and, enormously, the amount of sweat. Hence the great loss of weight. It stimulates also sexual appetite, though later on, power is lost whilst desire remains. After each injection the pupil dilates, but remains dilated only because injections are continued. As regards the brain, mental processes seem quickened, but a kind of hypnosis intervenes, so that the brain works without, and even against, the will. Immediately after the injection the cocainist becomes excited, and remains restless whilst under the influence. He likes manual work, however trifling, but has neither will nor ability for mental work, because he is bound to inject every five or ten minutes, or in fact, because he never ceases to inject. The hallucinations and illusions already mentioned make their appearance early. One syringe self-injected is, in my opinion, absolutely sure to produce the fascinating desire for a second. The individual is almost certainly then a cocainist, and will procure the drug for self-administration, even when apparently it is impossible to do so. All watching is useless. He has thousands of excuses to get a moment to himself, generally in the neighborhood of some chemist. Unscrupulous—even though still aware to some extent of his ties—he will get it dishonestly if necessary; and even when not craving for it at the moment, he will get it, because his only idea is to have it with him. The sense of right and wrong is not abolished, but he does not care much about trifles. Thus he sinks lower and lower, disregards his personal appearance, and because they will always show, or sham to show, a certain respect to his higher education, he seeks the association of lower people. He thus becomes a scoundrel or criminal, and does not mind to do so so long as he gets his cocaine. It is extremely seldom that he makes a trial to free himself of the habit, mainly because he does not see any reason to do so. Suicide he never contemplates so long as he can get his beloved drug."

